# The Diagnostic Value of the Cystoscope and Ureteral Catheter

**Published:** 1906-12

**Authors:** 


THE DIAGNOSTIC VALUE OF THE CYSTOSCOPE AND URETERAL CATHETER.—John Edgerton Cannaday concludes that the cystoscope as an accurate scientific instrument if of comparatively recent inception and perfection; that the other mechanical aids to the collection of the separate urines are not by any means absolutely dependable; that the cystoscope and ureteral catheter have their limitations, shortcomings and defects, and are not ideal; that these appliances are of especial value in diagnosing the causes of hematuria, in the discovery of calculi and foreign bodies in the bladder; that a careful study of the ureteral offices gives more than an inkling of the conditions present in ureter and kidney. Definite information concerning the origin of a pyuria ca be elicited by the use of the cystoscope and ureteral catheter which could not have been obtained in any other way.—*Medical Record,* November 24, 1906.

